# Correlation between serum trimethylamine-N-oxide and body fat distribution in middle-aged and older adults: a prospective cohort study

**DOI:** 10.1186/s12937-024-00974-w

**Published:** 2024-07-09

**Authors:** Si Chen, Xiao-yan Chen, Zi-hui Huang, Ai-ping Fang, Shu-yi Li, Rong-zhu Huang, Yu-Ming Chen, Bi-xia Huang, Hui-lian Zhu

**Affiliations:** 1 Department of Nutrition, Guangdong Provincial Key Laboratory of Food, Nutrition and Health, School of Public Health, Sun Yat-Sen University, 74 Zhongshan II Road, Guangzhou, 510080 PR China; 2 Department of Epidemiology, Guangdong Provincial Key Laboratory of Food, Nutrition and Health, School of Public Health, Sun Yat-Sen University, Guangzhou, China

**Keywords:** Serum TMAO, Fat distribution, Community-dwelling adults, Cohort study

## Abstract

**Background:**

Trimethylamine-N-oxide (TMAO) is linked with obesity, while limited evidence on its relationship with body fat distribution. Herein, we investigated the associations between serum TMAO and longitudinal change of fat distribution in this prospective cohort study.

**Methods:**

Data of 1964 participants (40–75y old) from Guangzhou Nutrition and Health Study (GNHS) during 2008–2014 was analyzed. Serum TMAO concentration was quantified by HPLC–MS/MS at baseline. The body composition was assessed by dual-energy X-ray absorptiometry at each 3-y follow-up. Fat distribution parameters were fat-to-lean mass ratio (FLR) and trunk-to-leg fat ratio (TLR). Fat distribution changes were derived from the coefficient of linear regression between their parameters and follow-up duration.

**Results:**

After an average of 6.2-y follow-up, analysis of covariance (ANCOVA) and linear regression displayed women with higher serum TMAO level had greater increments in trunk FLR (mean ± SD: 1.47 ± 4.39, *P*_*-trend*_ = 0.006) and TLR (mean ± SD: 0.06 ± 0.24, *P*_*-trend*_ = 0.011). Meanwhile, for women in the highest TMAO tertile, linear mixed-effects model (LMEM) analysis demonstrated the annual estimated increments (95% CI) were 0.03 (95% CI: 0.003 – 0.06, *P* = 0.032) in trunk FLR and 1.28 (95% CI: -0.17 – 2.73, *P* = 0.083) in TLR, respectively. In men, there were no similar significant observations. Sensitivity analysis yielded consistent results.

**Conclusion:**

Serum TMAO displayed a more profound correlation with increment of FLR and TLR in middle-aged and older community-dwelling women in current study. More and further studies are still warranted in the future.

**Trial registration:**

NCT 03179657.

**Supplementary Information:**

The online version contains supplementary material available at 10.1186/s12937-024-00974-w.

## Background

Obesity, characterized by a high body mass index (BMI), is linked to various health issues like metabolic syndrome (MetS) [1], and cardiovascular diseases (CVDs) [2]. However, the paradox that older adults with higher BMI tend to live longer, and that the aging population faces equal risks of chronic diseases regardless of BMI, has shifted focus to body fat distribution [3]. Specifically, the buildup of visceral adipose tissue (VAT) is strongly connected to problems like dyslipidemia, insulin resistance, and higher mortality risk, regardless of overall body weight or fat mass [4–6]. Additionally, the trunk-to-leg fat ratio (TLR), a marker of unfavorable fat distribution, is a valuable predictor of CVD mortality [7]. These changes in body composition are important aspects of aging and can harm overall health as individuals age [8].

Although sex hormones, aging, and genetic variations have been suggested as factors influencing body fat redistribution, studies on serum metabolites that could act as potential biomarkers for body fat distribution are limited. Trimethylamine-N-oxide (TMAO), a gut-derived metabolite produced following ingestion of dietary choline or carnitine, is regulated by these aforementioned factors [9]. Meanwhile, TMAO is reported to promote dyslipidemia, atherosclerosis, and clotting, making it an independent risk factor for CVD [10]. Researches also shown strong links between TMAO and obesity or obesity-related diseases [11–13]. Notably, a case–control study showed a significant positive correlation between serum TMAO and visceral fat in hemodialysis patients [14]. Meanwhile, participants with obesity in two different intervention programs demonstrated positive associations between serum TMAO levels and visceral fat mass or trunk fat at baseline [15, 16]. However, previous studies have often focused on individuals with hemodialysis or obesity, neglecting the general population, and primarily considered body weight or absolute fat quantity. What is more, whether serum TMAO levels relate to long-term unfavorable fat accumulation in the broader population remains uncertain. Therefore, further prospective studies are necessary to explore the link between serum TMAO levels and fat distribution, especially the adverse kind, which could inform strategies for preventing obesity and related metabolic conditions.

In this prospective cohort study, we enrolled community-dwelling Chinese adults aged 40 to 75 from the "Guangzhou Nutrition and Health Study (GNHS)" with a median follow-up of 6.2 years. Our goal was to investigate how serum TMAO levels relate to changes in fat distribution over time, focusing particularly on unfavorable fat distribution.

## Materials

### Participants

**Fig. 1:**
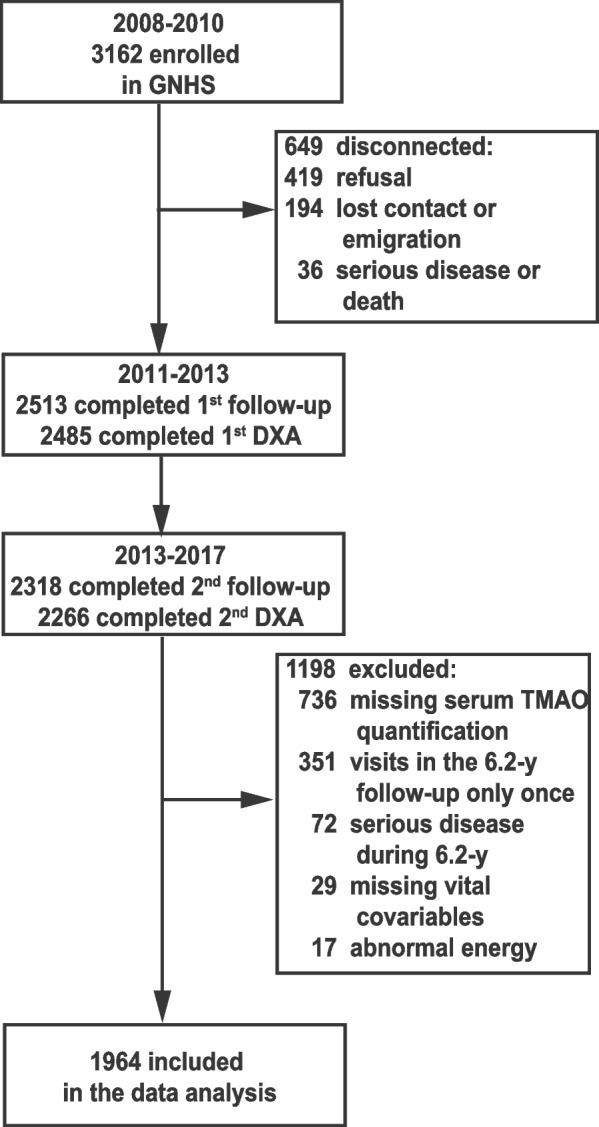
Flow chart of recruitment of participants

The data of this prospective cohort study were obtained from the Guangdong Nutrition and Health Study (GNHS), a prospective cohort study designed to explore the effects of dietary and environmental factors on cardio metabolic outcomes and osteoporosis. Recruitment took place from July 2008 to June 2010. As previously described [17], the inclusion criteria encompassed individuals aged 40 to 75 years who had resided in Guangzhou for at least five years. Demographic characteristics and biological samples were obtained at baseline; body composition was assessed during the follow-up phase every 3 years thereafter. The current exclusion criteria comprised: (1) self-reported or diagnosed cognitive impairment, immobility, chronic renal dysfunction, or cancer (*n* = 72) at baseline or during the 6.2-year follow-up period; (2) missing data on key covariates (*n* = 29); (3) implausible dietary energy intake (men, < 800 kcal/d or > 4000 kcal/d; women, < 500 kcal/d or > 3500 kcal/d) (*n* = 17); (4) missing data on serum TMAO level or serum TMAO level being out of 95% CI (*n* = 736); (5) participants did not complete two follow-up body composition examinations (*n* = 351). Eventually, a total of 1964 participants with a median follow-up of 6.2 years were included for the data analysis (Fig. 1). All participants provided written informed consent. The study protocol was approved by the ethics committee (L2017-004).

### Serum TMAO and its precursors measurement

After a 10-h fasting period, a 10-mL venous blood sample was collected at the beginning of the study and stayed undisturbed. S Serum was subsequently separated by centrifugation at 3000 × g for 15 min and stored at -80°C until analysis.

Serum TMAO and its precursors (choline, betaine) were quantified by high-performance liquid chromatography-tandem mass spectrometry (HPLC–MS/MS, Agilent 6400 Series Tridple Quad LC/MS, CA, USA), following previously established protocols [11]. In brief, 50 μL of serum was combined with a working solution containing internal standards (TMAO-d9, choline-d9, betaine-d9). Subsequently, 50 μL of acetonitrile spiked with 1% formic acid was added to the mixture and vortexed. After centrifugation at 15,100 × g at 4 °C for 10 min, approximately 120 μL of the supernatants were transferred into vials for HPLC–MS/MS analysis. The analytes and their corresponding isotopes were monitored using specific precursor-product ion transitions, with an iron spray voltage of 3500 V. The coefficients of variance (CVs) for between-run assays were 6.0% for TMAO, 4.91% for choline, and 6.21% for betaine.

### Body composition assessment

During each follow-up visit, a trained physician conducted an examination of the body composition of every participant. This assessment utilized dual-energy X-ray absorptiometry (DXA, Discovery W, Hologic Inc, Waltham, USA), operating in the "whole-body scans" mode.

The software accompanying the Hologic Discovery system (version 3.2) facilitated the direct acquisition of fat mass (FM) and bone mineral content (BMC). Absolute lean mass (LM) was determined by subtracting BMC from the original fat-free lean mass, thereby isolating non-bone lean mass. Precise positioning of regions of interest, such as the arms, legs, and trunk areas, and subsequent DXA scan analyses, were carried out by a proficient physician (Additional Fig. S1). Subsequently, to account for variations in weight, FM and LM were adjusted at baseline using the residual method. This adjustment was made in preparation for subsequent analyses [18].

### Covariates

The covariates were collected at baseline. Sociodemographic information (age and income), lifestyle (smoking, alcohol or tea drinking, and daily activity), and medical history were collected using a structured questionnaire administered in a face-to-face interview. Dietary intake was assessed by a validated 79-item food frequency questionnaire (FFQ) and adjusted by the energy residual method. Daily activities were assessed by a 19-item questionnaire, including daily occupation, leisure-time activity, and household chores [19]. Anthropometric data, including weight, height, and waist circumference (WC), were measured twice at baseline and each follow-up visit, and the averages were applied to later analyses. Fasting glucose levels were examined using fingertip blood sampling. Serum total cholesterol (TC), triglycerides (TG), and fasting glucose levels were quantified by colorimetric methods using a Roche Cobas 8000 C702 automated analyzer (Roche Diagnostics, Shanghai, China). The intra-assay coefficient of variation was 3.1% for TC, 5.8% for TG, and 2.5% for glucose, respectively [20]. For the concentration of serum TMAO would be affected by its precursors, the serum choline and betaine were also adjusted in the models.

### Statistical analysis

Descriptive data were expressed as either mean and standard deviation (SD) for normally distributed variables or median and interquartile range for non-normally distributed variables. Categorical variables were presented as frequencies and percentages. Given the observed differences in body composition between females and males, we displayed all primary results separately for women and men. Baseline characteristics were assessed based on data distribution, utilizing independent t-tests, Kruskal–Wallis tests, or χ^2^ tests.

The ratio of fat-to-lean mass (FLRs) or percentage of fat mass (FM%) for the entire body and specific regions were calculated. The trunk-to-leg fat ratio (TLR) was determined by the ratio of absolute trunk FM to leg FM. These FLR and TLR values served as indices for fat distribution (FD). Changes in FD (ΔFD) were derived from linear regression coefficients between these indices and follow-up duration, multiplied by the mean follow-up duration of 6.2 years [21].

Analysis of covariance (ANCOVA) was conducted to compare the mean 6.2-year changes across serum TMAO tertiles. This model was adjusted for baseline variables, including age, BMI, TG, TC, high-density lipoprotein cholesterol (HDL-C), low-density lipoprotein cholesterol (LDL-C), TMAO precursors (choline, betaine), dietary intake (energy, protein, and fat), lifestyle, and daily activity [5]. Preliminary results did not show significant differences in ΔFM across serum TMAO tertiles (all *P* > 0.05, Additional Tables S1 and S2); therefore, subsequent analyses focused on the association between serum TMAO and the 6.2-year ΔFD.

Multiple linear regressions (MLR) were employed to explore the adjusted association between ΔFD and each SD increment of serum TMAO. To validate these findings, linear mixed-effects models (LMEMs) were utilized to compare estimated changes in FD over time across serum TMAO tertiles. This analysis incorporated an interaction test between tertiles and time to account for repeated assessments of whole or regional FD over time.

Sensitivity analysis was performed on participants under 65 years of age who had complete covariate data. For participants with missing covariate values, multivariate imputation by chained equations (MICE) was employed to address these gaps. All statistical tests were two-sided, and statistical significance was determined at a *P*-value < 0.05. Statistical analyses were conducted using R software (version 4.1.2). LMEMs were analyzed using the "nlme" package (version 3.1–152), MICE was conducted using "mice" (version 3.13.0), and additional packages included "tidyverse" (version 1.3.1) and "VIM" (version 6.1.1) in R studio (version 1.3.1093). Data visualization was achieved using the "ggplot2" package (version 3.3.5) in R studio.

## Results

### Characteristics of the study participants

A total of 1964 eligible participants, consisting of 1423 women and 541 men, were enrolled in the study. The sex-specific baseline characteristics of the participants are detailed in Table 1**.** The median age of all participants at baseline was 57 years, and the average serum TMAO level was 2.33 μmol/L. In comparison to women, men were older and had higher monthly income levels (> 3000 yuan/month). Additionally, men exhibited a higher likelihood of developing CVDs, type 2 diabetes, or obesity. Furthermore, men engaged in less physical activity and had lower levels of TC, HDL-C, LDL-C, and TG (all *P* < 0.05)**.**

**Table 1:** Baseline characteristics of participants

	Total	Women	Men	*P*
N	1964	1423	541	
serum TMAO, μmol/L^1^	2.3 ± 2.2	2.4 ± 2.3	2.3 ± 2.1	0.569
Age (years)^1^	57.4 ± 4.9	56.7 ± 4.6	59.1 ± 5.1	< 0.001
Post-menopause (n, %)^3^	1112 (78.1)	1112 (78.1)	-	
Monthly income (Yuan/person)^3^				0.002
< 1500	628 (32.7)	460 (33.0)	168 (32.1)	
1500–3000	909 (47.4)	669 (47.9)	240 (45.9)	
> 3000	382 (19.9)	267 (13.9)	115 (22.0)	
Dietary intakes				
Energy (kcal/d)^2^	1759.6 (1494.4, 2129.3)	1680.0 (1434.5, 2025.8)	2031.9 (1661.4, 2370.0)	< 0.001
Protein (g/d)^2^	73.5 (60.0, 90.6)	70.8 (58.4, 87.2)	81.2 (66.5, 98.3)	< 0.001
Fat (g/d)^2^	57.5 (44.3, 75.4)	55.2 (42.3, 72.9)	65.0 (50.0, 81.5)	< 0.001
Daily Activity (MET)^2^	36.5 (30.5, 58.2)	36.6 (30.7, 58.0)	36.1 (30.1, 58.4)	< 0.001
Alcohol drinker (n, %)^3^	101 (5.2)	28 (1.4)	73 (13.7)	< 0.001
Tea drinker (n, %)^3^	972 (50.1)	607 (43.1)	365 (68.7)	< 0.001
Smoking (n, %)^3^	262 (13.4)	7 (0.5)	255 (47.1)	< 0.001
BMI (kg/m^2^)^1^	23.2 ± 0.1	23.0 ± 0.2	23.8 ± 0.1	< 0.001
Waist-to-hip ratio (WHR)^1^	0.88 ± 0.05	0.87 ± 0.06	0.92 ± 0.05	< 0.001
Obesity (n, %)^3^	708 (36.0)	473 (33.2)	235 (43.4)	< 0.001
CVDs (n, %)^3^	194 (11.5)	159 (13.0)	35 (7.7)	0.007
Type 2 diabetes (n, %)^3^	76 (4.5)	45 (3.7)	31 (6.8)	0.032
Hypertension (n, %)^3^	347 (20.6)	246 (20.0)	101 (22.1)	0.281
Biochemical indicators				
TC, mmol/L^2^	5.4 (4.8, 6.1)	5.6 (4.9, 6.20)	5.0 (4.4, 5.7)	< 0.001
HDL-C, mmol/L^2^	1.4 (1.2, 1.6)	1.4 (1.2, 1.7)	1.3 (0.9, 1.9)	0.039
LDL-C, mmol/L^2^	3.6 (3.0, 4.2)	3.6 (3.1, 4.2)	3.4 (2.9, 4.0)	< 0.001
TG, mmol/L^2^	1.3 (0.9, 1.8)	1.3 (0.9, 1.8)	1.0 (0.7, 1.7)	0.020
Fasting Glu, mmol/L^2^	4.6 (4.0, 5.0)	4.6 (4.2, 5.0)	4.7 (4.3, 5.1)	0.001
betaine, μmol/L^1^	51.3 ± 0.4	49.8 ± 16.7	55.4 ± 0.7	< 0.001
choline, μmol/L^1^	21.9 ± 0.4	21.1 ± 15.1	23.9 ± 0.7	0.001

*TMAO* trimethylamine N-oxide, *BMI* body mass index, *WHR* waist-to-hip ratio, *CVDs* cardiovascular diseases, *TC* total cholesterol, *HDL-C* high-density lipoprotein cholesterol, *LDL-C* low-density lipoprotein cholesterol, *TG* triglycerides, *MET* metabolic equivalent-h/d, *IQR* interquartile range

^1^Normally distributed continuous data are presented as mean with SD. T-test was conducted for calculating the *P*-value

^2^Non-normally distributed continuous data are presented as median with IQR. Kruskal–Wallis was conducted for the *P*-value

^3^Categorical data are presented as frequency and percentage. χ^2^ tests were conducted for calculating the *P*-value

### Changes in fat distribution over 6.2 years among serum TMAO level tertiles

**Fig. 2:**
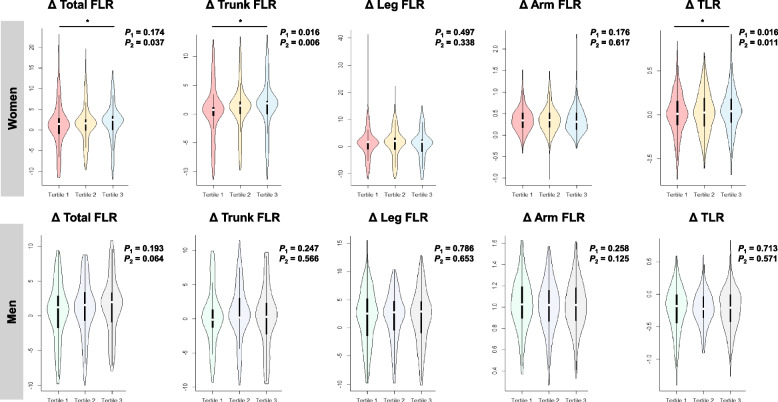
Comparisons of Δ FD indices across serum TMAO tertiles are shown by violin plot. *P*_1_:ANCOVA were conducted to compare the difference in total and regional Δ FD indices across the tertiles of serum TMAO after adjusted co-variables of baseline age, BMI, dietary intake (energy, protein, and fat), daily activity, and lifestyles (smoking, alcohol drinking), TC, TG, HDL-C, LDL-C, serum choline, and betaine. *P*_2_: Median of each serum TMAO tertile was applied for p-trend test using linear regression model to investigate the dose-response relationship of total and regional Δ FD indices across the tertiles of serum TMAO. The value of* P*_2_ was obtained after adjusting covariates of baseline age, BMI, dietary intake (energy, protein, and fat), daily activity, and lifestyles (smoking, alcohol drinking), TC, TG, HDL-C, LDL-C, serum choline, and betaine

To investigate differences in 6.2-year ΔFD across serum TMAO tertiles, we employed ANCOVA analyses. As illustrated in Fig. 2, after adjusting for potential confounding factors, women exhibited a dose–response relationship in Δ total FLR, Δ trunk FLR, and Δ TLR across serum TMAO level tertiles (*P*_*trend*_ = 0.037, 0.006, and 0.011, respectively). However, no significant differences in Δ FD were observed among men (Fig. 2).

Subsequently, MLR analysis was conducted to explore associations between each SD increase of serum TMAO levels and ΔFD. In women, the results showed that each SD increase in serum TMAO level (0.44 μmol/L) was associated with a 0.260 increase in Δ trunk FLR (*P* = 0.030) and a 0.069 increase in in ΔTLR (*P* = 0.024) (Additional Table S3). Similar associations were observed when analyzing TMAO as a continuous variable (Additional Fig. S2). However, in men, no significant associations were observed between each SD increase in serum TMAO levels (0.42 μmol/L) and any of the 6.2-year Δ FD indices (all *P* > 0.05, Additional Table S3 and Fig. S3).

To confirm the association between FD and serum TMAO levels over time, LMEMs were employed (Table 2). After adjusting for covariates, the interaction analysis indicated that in women, within the highest tertile of serum TMAO level, there were estimated mean changes (95% CI) per year of 1.28 (-0.17, 2.73)/year in trunk FLR and 0.03 (0.003, 0.06)/year in TLR (*P*_-interaction_ = 0.083 and 0.032, respectively). Conversely, no significant associations were observed in men (Table 2).

**Table 2:** Linear mixed model for the association of serum TMAO level with total or regional Δ FD parameters over the 6.2-year follow-up

	Women	***P***	Men	***P***
Total FLR (ref. Tertile 1)				
Tertile2	-1.14 (-3.86, 1.57)	0.409	-1.15 (-4.27, 1.97)	0.471
Tertile3	0.38 (-2.34, 3.10)	0.786	0.56 (-2.59, 3.70)	0.729
Time	0.88 (-0.51, 2.27)	< 0.001	1.19 (-0.40, 2.77)	< 0.001
Tertile2*Time	0.66 (-1.06, 2.38)	0.423	2.18 (-1.75, 2.19)	0.828
Tertile3*Time	0.47 (-1.26, 2.20)	0.591	-0.56 (-2.54, 1.42)	0.577
Trunk FLR (ref. Tertile 1)				
Tertile2	1.10 (-1.26, 3.46)	0.361	-0.55 (-3.20, 2.10)	0.683
Tertile3	**2.67 (0.35, 4.99)**	**0.024**	0.29 (-2.38, 2.96)	0.832
Time	0.30 (0.29, 0.31)	< 0.001	-6.51 (-7.86. -5.17)	< 0.001
Tertile2*Time	0.64 (-0.84, 2.11)	0.397	-0.01 (-1.66, 1.69)	0.988
Tertile3*Time	1.28 (-0.17, 2.73)	0.083	-0.31 (-1.99, 1.37)	0.716
Leg FLR (ref. Tertile 1)				
Tertile2	-1.50 (-4.88, 1.89)	0.386	-1.28 (-4.39, 1.82)	0.418
Tertile3	0.06 (-3.34, 3.46)	0.974	-0.95 (-2.18, 4.08)	0.552
Time	0.36 (-1.37, 2.10)	< 0.001	1.73 (0.15, 3.31)	< 0.001
Tertile2*Time	0.70 (-1.45, 2.85)	0.524	0.46 (-1.52, 2.41)	0.657
Tertile3*Time	0.53 (-1.63, 2.68)	0.633	-0.65 (-2.63, 1.32)	0.517
Arm FLR (ref. Tertile 1)				
Tertile2	-2.51 (-7.22, 2.19)	0.296	-0.91 (-5.47, 1.65)	0.294
Tertile3	0.94 (-3.79, 5.66)	0.698	0.33 (-3.26, 3.91)	0.858
Time	0.94 (-3.79, 5.66)	< 0.001	3.69 (1.88, 5.50)	< 0.001
Tertile2*Time	1.23 (-1.76, 4.21)	0.421	0.50 (-1.75,2.75)	0.664
Tertile3*Time	0.26 (-2.74, 3.26)	0.865	-0.57 (-2.83,1.67)	0.624
Trunk-to-leg fat ratio (ref. Tertile 1)				
Tertile2	-0.02 (-0.07, 0.03)	0.611	0.05 (-0.09,0.19)	0.497
Tertile3	0.05 (-0.004, 0.10)	0.251	3e-04(-0.14, 0.14)	0.996
Time	1.98 (0.04,0.08)	< 0.001	-0.14(-0.21, -0.07)	< 0.001
Tertile2*Time	0.02 (-0.02, 0.04)	0.264	-0.03 (-0.12, 0.06)	0.496
Tertile3*Time	**0.03(0.003, 0.06)**	**0.032**	-8e-04 (-0.09,0.09)	0.985

The data of a total of 1423 women and 541 men were assessed using LMEMs with serum TMAO, time, and the serum TMAO × time interaction term as fixed effects and the subject identifier as a random intercept. All models were adjusted for baseline age, BMI, energy intake, protein intake, fat intake, alcohol consumption, smoking, tea drinking, physical activity, TC, TG, HDL-C, and LDL-C levels. The mean follow-up duration was 6.2-year on an average. LMEM, linear mixed-effects model

### Sensitivity analyses

To account for the impact of age on body fat mass, we conducted sensitivity analyses on participants under the age of 65 who had complete covariate data. In women, those in the highest tertile of serum TMAO still exhibited a greater increase in trunk FLR (*P*_*trend*_ = 0.019) and ΔTLR (*P*_*trend*_ = 0.087) compared to those in the lowest tertile (Additional Table S4). LMEMs further demonstrated an annual increment of 0.02 in trunk FLR (*P* = 0.041) and and 2.91 in TLR (*P* = 0.012) among women (Additional Table S5). However, similar to previous analyses, no significant associations between TMAO and changes in Δ FD were found in the sensitivity analysis.

For women, we further investigated whether menopausal status influences the distribution of body fat. Our findings show that the correlations between serum TMAO levels and trunk FLR and the change in ΔTLR were only significant in postmenopausal women (Additional Fig. S4).

## Discussion

In this study, we examined the correlation between serum TMAO and long-term fat distribution in community-dwelling adults aged 40–75. In the follow-up period with a median of 6.2 years, we discovered that higher serum TMAO levels were significantly associated with increased FLR and TLR, especially in middle-aged and older women.

As individuals age, changes occur in their body weight and composition. Previous studies have mainly emphasized the strong correlation between higher body weight, often measured using BMI, and the risk of obesity-related diseases and CVDs [22–24]. However, recent research has drawn attention to the role of body fat distribution in these conditions. For example, a 12-year follow-up study found that body fat distribution was independently associated with cardiovascular disease risk in participants from the UK biobank [25]. Another cross-sectional study found that the ratio of trunk fat to peripheral fat, as determined by DXA examination, was positively associated with CVDs, independent of total body fat mass [26]. These findings indicate that BMI alone is insufficient as a predictor of these diseases, emphasizing the importance of monitoring changes in both body fat mass and distribution as individual age.

TMAO, a potentially harmful metabolite originating in the gut, is produced when dietary choline or carnitine is oxidized by the liver enzyme flavin monooxygenase 3 (FMO3) [27]. Previous research has identified TMAO as an independent risk factor for the CVD [10], and it is associated with insulin resistance and obesity [28, 29] However, a recent meta-analysis revealed a nonlinear correlation between TMAO levels and BMI in healthy individuals [13]. The existence of the obesity paradox during the natural aging process further highlights complexity of the relationship between TMAO levels and body weight (BMI) as well as body fat distribution in assessing chronic disease risk [30].

In the current study, we observed that changes in trunk FLR and TLR (Fig. 2**, **Table 2) increased with higher serum TMAO levels, particularly among women. Notably, the absolute body fat mass did not show significant changes (Additional Tables S1 and S2). Both FLR and TLR are well-established predictors of CVDs [5, 31]. Fat distribution in the trunk and legs can be influenced by factors such as inflammation and insulin resistance [32, 33]. In a study involving healthy Mexican adolescents, a positive association was found between trunk FLR and hyperinsulinemia [34]. Furthermore, the Korea National Health and Nutrition Examination Survey revealed a significant association between higher trunk FLR and increased prevalence of MetS [35]. While there is limited direct epidemiological evidence linking metabolically unhealthy fat distribution to TMAO, there are indications that TMAO could potentially impact body composition, specifically influencing fat distribution in the trunk and legs. A recent study demonstrated that TMAO exacerbated sarcopenic obesity development through ROS-AKT/mTOR signaling in aged mice fed a high-fat diet [36]. Additionally, previous reports have shown that elevated circulating TMAO could induce low-grade chronic systemic inflammation through various inflammatory proteins (IL-6, ICAM1, COX2, LPS etc.) and contribute to metabolic dysfunction [37, 38]. Moreover, a meta-analysis concluded that TMAO may serve as a novel biomarker for diabetes mellitus, CVDs, and kidney function [39]. These findings suggest that serum TMAO levels may impact fat distribution in the trunk and legs during the aging process, emphasizing the need for further research to fully comprehend the underlying mechanisms.

The present study demonstrated a sex-related difference in the association between changes in body fat distribution and serum TMAO levels. One plausible explanation for this sex-related difference lies in the regulation of FMO3 expression by sex hormones. FMO3, responsible for the majority of hepatic TMA conversion to TMAO, exhibits higher expression in the female liver compared to the male liver [40]. Notably, the majority of women in our study were postmenopausal with an average age of 57, and it has been reported that declining estrogen levels might down-regulate FMO3 activation [40]. In our stratified analysis (Additional Fig. S4), we observed that only postmenopausal women showed increased trunk FLR (*P* = 0.009) and ΔTLR (*P* < 0.001) with elevated serum TMAO levels. However, the proportion of premenopausal women in the analysis was merely 22.9%, which could explain the lack of significance in the interaction effects between serum TMAO and menopausal status. Furthermore, as women age, the composition of their gut microbiome tends to shift towards a profile abundant in *Firmicutes*, leading to elevated levels of secondary bile acids and activation of the farnesoid X receptor (FXR) [41]. FXR, a crucial regulator of FMO3, may counterbalance the effects of reduced estrogen levels, partially elucidating the sex-related distinctions. Additionally, FMO3 contributes to metabolic dysfunction through TMAO, which triggers PERK signaling [38]. Studies in humans and rodents have consistently shown that estrogens promote fat accumulation in subcutaneous adipose tissue in women rather than visceral adipose tissue (VAT). The decrease in estrogen levels post-menopause contributes to increased visceral fat mass and metabolic obesity [42]. In contrast, adult men typically have a lower average total body fat percentage compared to adult women due to testosterone, despite a tendency for greater fat accumulation in VAT. These findings imply that women may be more vulnerable to the effects of serum TMAO concerning fat distribution.

The study has several strengths. Firstly, it is the first to investigate the link between serum TMAO levels and unhealthy fat accumulation in middle-aged and older Chinese adults using a prospective study design. Secondly, the study had a relatively large sample size and considered potential socioeconomic and behavioral factors that could affect the association between serum TMAO levels and body composition. Thirdly, a comparison between participants included and excluded from the study (Additional Table S6) showed that those included had healthier lifestyles, with fewer smokers, less alcohol consumption, and lower incomes. Despite the potential underestimation of results, the associations between serum TMAO levels and central fat deposition remained robust after adjusting for these factors. Additionally, accurate body composition measurements were obtained from participants using DEXA [43]. The quantification of Serum TMAO levels was precise, rapid, and stable using HPLC–MS.

However, this study had some limitations. The findings may not be directly applicable to other populations because this was a single-center study. A single assessment of TMAO levels did not account for changes in TMAO levels over time, so the LMEM analysis was performed to minimize the impact of covariates on TMAO and body fat over time. The smaller sample size of men in the study may have contributed to the lack of significant findings in this group, emphasizing the need for larger studies involving men in the future. Moreover, future research should consider measuring sex hormones to better assess post-menopausal status. Finally, it's important to note that since the study was observational in nature, establishing causality is not possible.

## Conclusion

In conclusion, the study found a positive association between serum TMAO levels and long-term increases in trunk fat-to-lean ratio (FLR) and trunk-to-limb fat ratio (TLR) in Chinese women aged 40 to 75 years living in the community. This discovery suggests a potential strategy for preventing obesity or sarcopenia during aging. However, further research is needed to explore the underlying mechanisms of this association.

### Supplementary Information

Supplementary Material 1.

Supplementary Material 2.

## Data Availability

No datasets were generated or analysed during the current study.

## Abbreviations

TMAO: Trimethylamine N-oxide
GNHS: Guangdong Nutrition and Health Study
HPLC–MS/MS: High-performance liquid chromatography-tandem mass spectrometry
DXA: Dual energy X-ray absorptiometer
FM: Fat mass
LM: Lean mass
FLR: Fat-mass to lean-mass ratio
TLR: Trunk-to-leg fat ratio
VAT: Visceral adipose tissue
BMI: Body mass index
WHR: Waist-to-hip ratio
MetS: Metabolic syndrome
CVDs: Cardiovascular diseases
TMA: Trimethylamine
FMO3: Flavin-containing monooxygenase 3
BMC: Bone mineral content
FFQ: Food frequency questionnaire
WC: Waist circumference
HDL-C: High density leptin cholesterol
LDL-C: Low density leptin cholesterol
TC: Total cholesterols
TG: Triglycerides
ANOVA: Analysis of variance
LMEMs: Linear mixed-effects models
MICE: Multivariate imputation by chained equations
MET: Metabolic equivalent
SCAT: Subcutaneous adipose tissue
