# Supplementary material for: Correlation between serum trimethylamine-N-oxide and body fat distribution in middle-aged and older adults: a prospective cohort study
Source: Nutr J. 2024 Jul 9;23:70. doi: 10.1186/s12937-024-00974-w (PMC11234726; doi:10.1186/s12937-024-00974-w)
Supplement: Supplementary file 1 — Supplementary Material 1. [file 12937_2024_974_MOESM1_ESM.docx]

**Additional Figures**


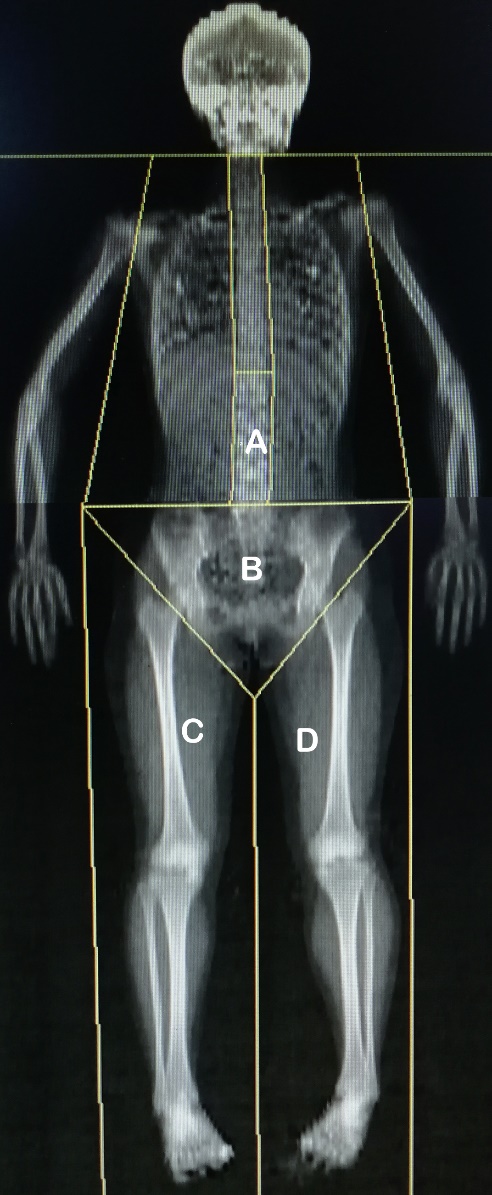
**FigS1**

**FigS1** **Analysis of total and regional whole-body scan using the DXA according to previous study description [14]**. For the picture of the scan could not be completely exported, we merged two parts of a participant scan picture. Briefly, the trunk is considered as the region delimited by a horizontal line passing under the chin, two vertical lines passing through the medial margin of the head of the humerus, excluding all of the upper limbs, and two oblique lines at the groin cutting midway through the neck of the femur and crossing below the pubis. The trunk-to-leg ratio was calculated as the ratio of the FM-trunk to the FM-legs: (A+B)/(C+D).

**
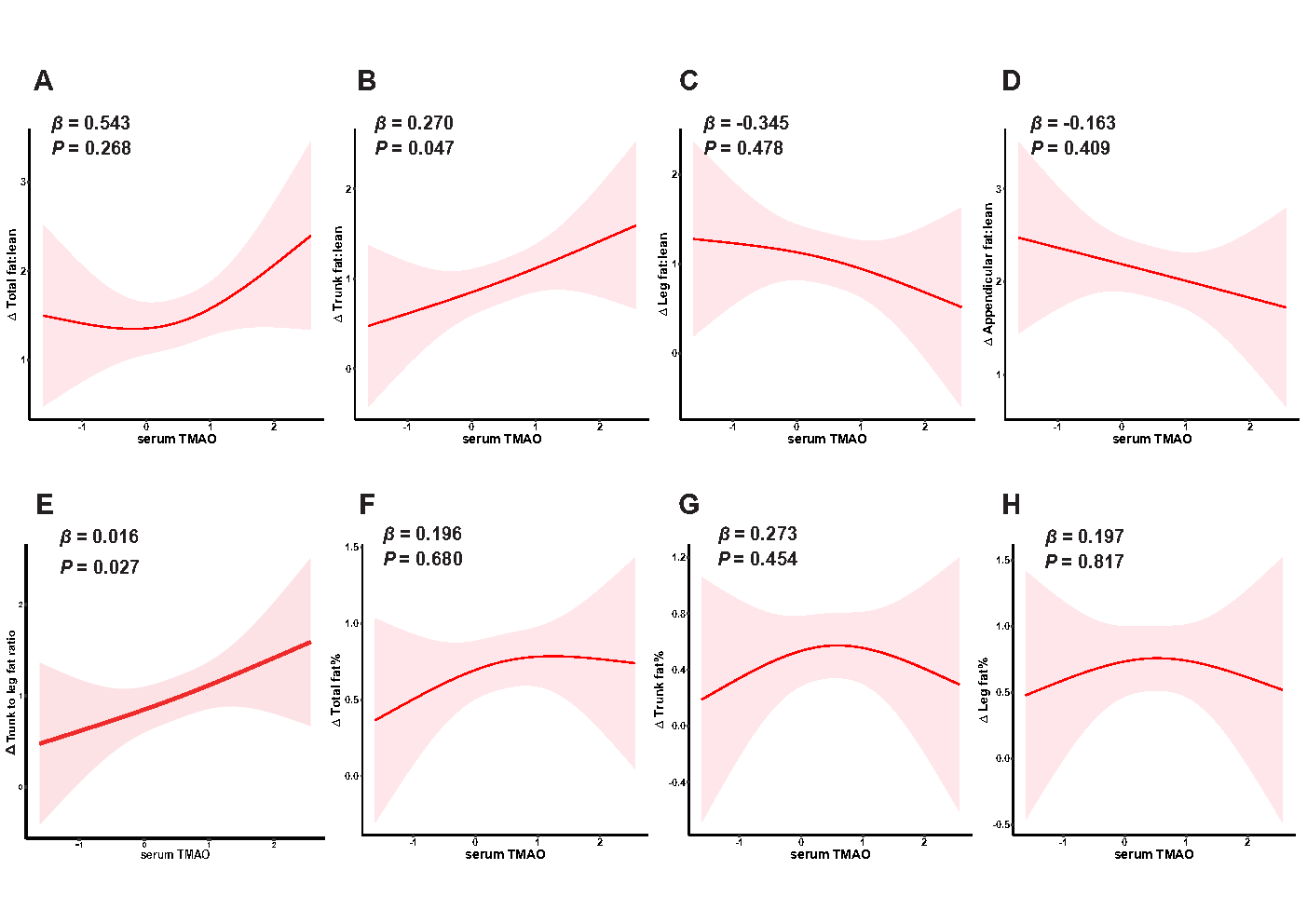
FigS2**

**FigS2 Adjusted associations between baseline TMAO and ΔFD indices in women.**

The mean of the duration of follow-up was 6.2 y on average. Regional Δ body fat mass or distribution indices were represented as the coefficients of linear regression between body composition and follow-up duration multiplied by 6.2-y. Serum TMAO was ln-transformed.

Relationships between serum TMAO and **(A)** Δ total FLR (*β =* 0.543, *P =* 0.268) **(B)** Δ trunk FLR mass (*β =* 0.270, *P =* 0.047) **(C)** Δ leg FLR mass (*β =* -0.345, *P =* 0.478) **(D)** Δ appendicular FLR mass (*β =* -0.163, *P =* 0.409) are depicted.

**(E)** Picture displays relationship between serum TMAO and Δ TLR (*β =* 0.016, *P =* 0.027).

**(F-H)** Pictures demonstrates associations between serum TMAO and Δ FM% in whole-body (*β =* 0.196, *P =* 0.680), trunk (*β =* 0.273, *P =* 0.454) and leg (*β =* 0.197, *P =* 0.817) respectively.


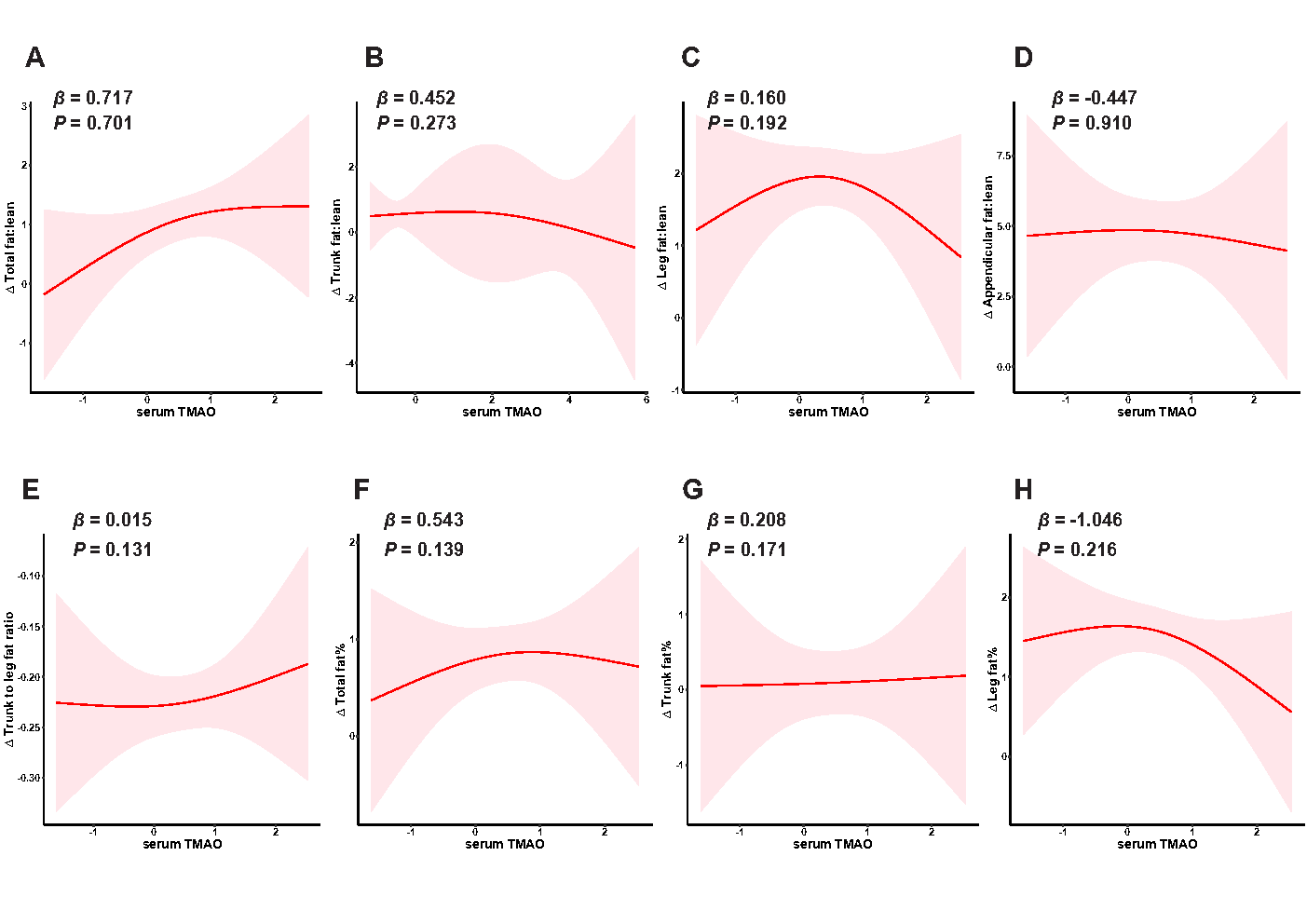
**FigS3**

**FigS3 Adjusted associations between baseline TMAO and Δ FD indices in men.**

The mean of the duration of follow-up was 6.2-y on average. Regional Δ body fat mass or distribution indices were represented as the coefficients of linear regression between body composition and follow-up duration multiplied by 6.2-y. Serum TMAO was ln-transformed.

Relationships between serum TMAO and **(A)** Δ total FLR (*β =* 0.717, *P =* 0.701) **(B)** Δ trunk FLR (*β =* 0.452, *P =* 0.273) **(C)** Δ leg FLR (*β =* 0.160, *P =* 0.192) **(D)** Δ appendicular FLR (*β =* 0.447, *P =* 0.910) are depicted.

**(E)** Picture displays relationship between serum TMAO and Δ TLR (*β =* 0.015, *P =* 0.131).

**(F-H)** Pictures demonstrates associations between serum TMAO and Δ FM% in whole-body (*β =* 0.543, *P =* 0.139), trunk (*β =* 0.208, *P =* 0.171) and leg (*β =* -1.046, *P =* 0.216) respectively.


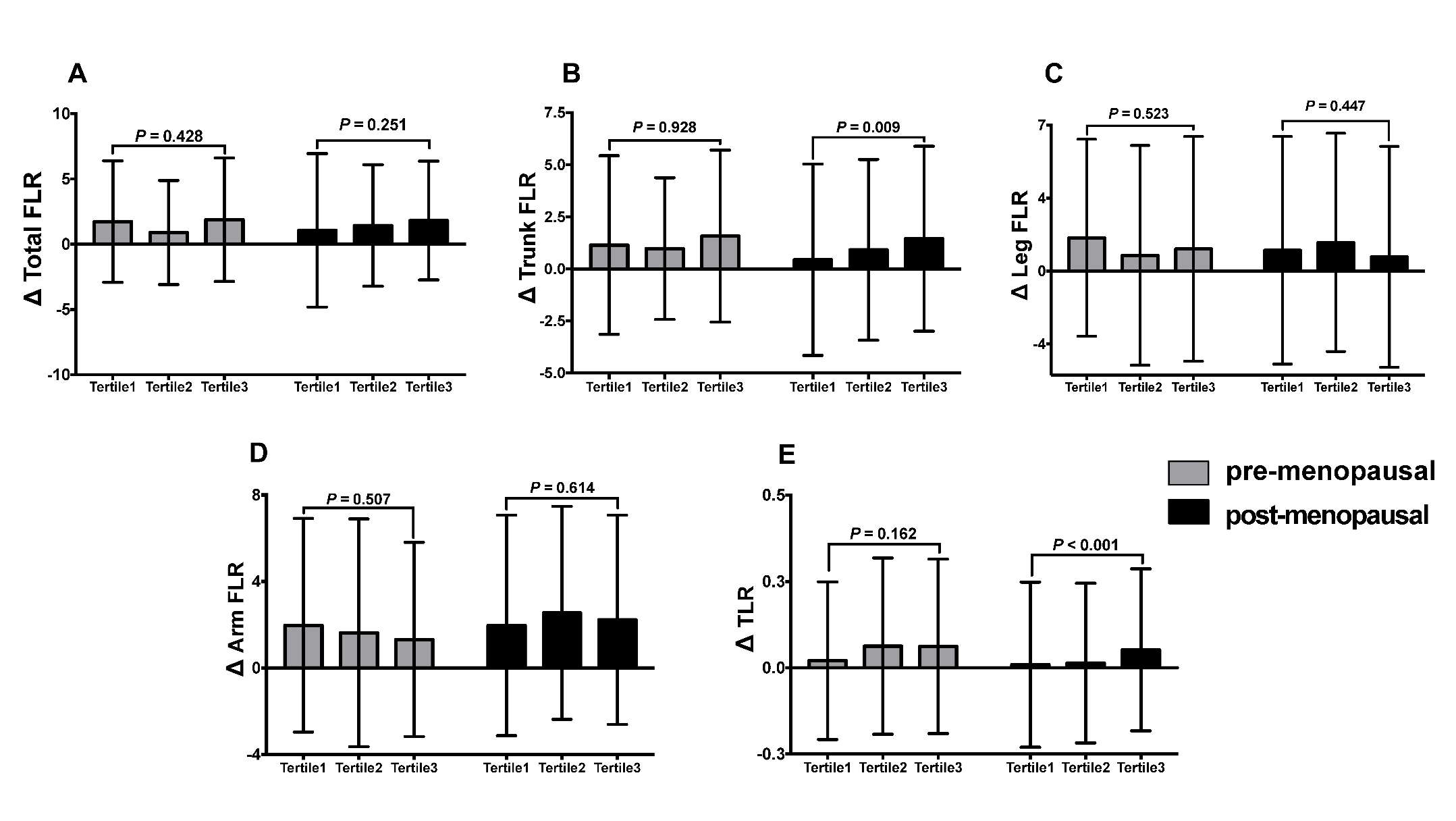
**FigS4**

**FigS4 Stratified analysis for comparing the Δ FD indices between pre- and post-menopausal women across baseline TMAO tertiles.** The results of ANCOVA analysis displayed the adjusted ratio of (**A**) Δ total FLR (*P*-interaction = 0.387); (**B**) Δ trunk FLR (*P*-interaction = 0.636); (**C**) Δ leg FLR (*P*-interaction = 0.170); (**D**) Δ appendicular FLR (*P*-interaction = 0.444); (**E**) Δ TLR (*P*-interaction = 0.416) across serum TMAO tertiles.
