# Supplementary material for: Correlation between serum trimethylamine-N-oxide and body fat distribution in middle-aged and older adults: a prospective cohort study
Source: Nutr J. 2024 Jul 9;23:70. doi: 10.1186/s12937-024-00974-w (PMC11234726; doi:10.1186/s12937-024-00974-w)
Supplement: Supplementary file 2 — Supplementary Material 2. [file 12937_2024_974_MOESM2_ESM.docx]

**Additional Tables**

**Table S1 Total and regional changes of absolute fat mass (Δ AFM) over 6.2-y among serum TMAO tertiles ***

|  | **Women** | | | | | **Men** | | | | |
| --- | --- | --- | --- | --- | --- | --- | --- | --- | --- | --- |
|  | **Tertile 1**  **(N = 474)** | **Tertile 2**  **(N = 479)** | **Tertile 3**  **(N = 470)** | ***P*_1_** | ***P*_2_** | **Tertile 1**  **(N = 180)** | **Tertile 2**  **(N = 181)** | **Tertile 3**  **(N = 180)** | ***P*_1_** | ***P*_2_** |
| TMAO (μmol/L) | 0.80 ± 0.44 | 1.65 ± 0.70 | 4.61 ± 2.76 |  |  | 0.80 ± 0.42 | 1.71 ± 0.61 | 4.35 ± 2.32 |  |  |
| Δ Total AFM (kg) |  |  |  |  |  |  |  |  |  |  |
| Model 1 | 0.32 ± 2.62 | 0.55 ± 2.73 | 0.38 ± 2.71 | 0.408 | 0.835 | 0.65 ± 2.96 | 0.73 ± 2.59 | 0.69 ± 2.92 | 0.962 | 0.588 |
| Model 2 | 0.34 ± 2.61 | 0.53 ± 2.76 | 0.39 ± 2.72 | 0.406 | 0.944 | 0.59 ± 2.87 | 0.70 ± 2.57 | 0.62 ± 3.00 | 0.903 | 0.457 |
| Δ Trunk AFM (kg) |  |  |  |  |  |  |  |  |  |  |
| Model 1 | -0.02 ± 1.57 | 0.07 ± 3.39 | 0.09 ± 1.77 | 0.526 | 0.789 | -0.13 ± 1.81 | -0.07 ± 1.70 | -0.06 ± 1.87 | 0.931 | 0.994 |
| Model 2 | -0.03 ± 1.58 | 0.04 ± 1.74 | 0.08 ± 1.79 | 0.382 | 0.715 | -0.17 ± 1.77 | -0.10 ± 1.69 | -0.13 ± 1.91 | 0.897 | 0.941 |
| Δ Leg AFM (kg) |  |  |  |  |  |  |  |  |  |  |
| Model 1 | 0.06 ± 1.11 | 0.09 ± 1.10 | 0.07 ± 1.04 | 0.925 | 0.960 | 0.39 ± 1.08 | 0.45 ± 0.94 | 0.45 ± 0.99 | 0.823 | 0.864 |
| Model 2 | 0.08 ± 1.11 | 0.08 ± 1.11 | 0.07 ± 1.04 | 0.997 | 0.640 | 0.37 ± 1.04 | 0.46 ± 0.96 | 0.43 ± 1.01 | 0.865 | 0.543 |
| Δ Arm AFM (kg) |  |  |  |  |  |  |  |  |  |  |
| Model 1 | 0.03 ± 0.49 | 0.04 ± 0.53 | 0.03 ± 0.62 | 0.732 | 0.871 | 0.33 ± 0.51 | 0.30 ± 0.36 | 0.30 ± 0.44 | 0.761 | 0.237 |
| Model 2 | 0.04 ± 0.48 | 0.04 ± 0.54 | 0.04 ± 0.62 | 0.623 | 0.646 | 0.34 ± 0.51 | 0.31 ± 0.36 | 0.32 ± 0.45 | 0.621 | 0.280 |

*: Change of absolute fat mass (ΔAFM) was annual change of AFM multiplied 6.2 years. Annual change of AFM was represented as the coefficients of linear regression between AFM and follow-up duration.

Data in model 1 were analyzed for the crude *P*-value across the tertiles of serum TMAO.

Data in model 2 were analyzed for the adjusted *P*-value across the tertiles of serum TMAO after adjusted covariates of baseline age, BMI, dietary intake (energy, protein and fat), daily activity and lifestyles (smoking, alcohol drinking), TC, TG, HDL-C, LDL-C, serum choline and betaine.

*P*_1_: Mean in each serum TMAO tertile was compared by ANOVA or ANCOVA for *P*_1_ to compare differences of total or regional ΔAFM across serum TMAO tertiles.

*P*_2_: Median of each serum TMAO tertile was applied in the linear regression model for *P*_trend_ to investigate the dose-response relationship of total and regional ΔAFM across the tertiles of serum TMAO.

**Table S2 Total and regional changes of percentage fat mass (FM%) over 6.2-y among serum TMAO tertiles ***

|  | **Women** | | | | | **Men** | | | | |
| --- | --- | --- | --- | --- | --- | --- | --- | --- | --- | --- |
|  | **Tertile 1**  **(N = 474)** | **Tertile 2**  **(N = 479)** | **Tertile 3**  **(N = 470)** | ***P*_1_** | ***P*_2_** | **Tertile 1**  **(N = 180)** | **Tertile 2**  **(N = 181)** | **Tertile 3**  **(N = 180)** | ***P*_1_** | ***P*_2_** |
| TMAO (μmol/L) | 0.80 ± 0.44 | 1.65 ± 0.70 | 4.61 ± 2.76 |  |  | 0.80 ± 0.42 | 1.71 ± 0.61 | 4.35 ± 2.32 |  |  |
| Δ Total FM % |  |  |  |  |  |  |  |  |  |  |
| Model 1 | 0.62 ± 3.14 | 0.71 ± 3.20 | 0.87 ± 3.26 | 0.480 | 0.503 | 0.55 ± 3.18 | 0.90 ± 3.22 | 1.06 ± 3.50 | 0.324 | 0.314 |
| Model 2 | 0.64 ± 3.16 | 0.66 ± 3.23 | 0.81 ± 3.31 | 0.647 | 0.602 | 0.48 ± 3.20 | 0.91 ± 3.22 | 0.97 ± 3.57 | 0.408 | 0.283 |
| Δ Trunk FM % |  |  |  |  |  |  |  |  |  |  |
| Model 1 | 0.41 ± 3.99 | 0.54 ± 4.47 | 0.67 ± 4.03 | 0.642 | 0.798 | -0.14 ± 4.96 | 0.01 ± 4.50 | 0.41 ± 4.81 | 0.540 | 0.501 |
| Model 2 | 0.45 ± 4.03 | 0.48 ± 4.47 | 0.62 ± 4.07 | 0.394 | 0.544 | -0.29 ± 4.93 | -0.03 ± 4.47 | 0.27 ± 4.87 | 0.434 | 0.363 |
| Δ Leg FM % |  |  |  |  |  |  |  |  |  |  |
| Model 1 | 0.63 ± 4.38 | 0.82 ± 4.42 | 0.76 ± 4.42 | 0.794 | 0.924 | 1.65 ± 3.30 | 1.61 ± 3.28 | 1.38 ± 3.56 | 0.717 | 0.370 |
| Model 2 | 0.61 ± 4.35 | 0.78 ± 4.42 | 0.73 ± 4.38 | 0.563 | 0.780 | 1.57 ± 3.31 | 1.68 ± 3.24 | 1.27 ± 3.62 | 0.461 | 0.243 |
| Δ Arm FM % |  |  |  |  |  |  |  |  |  |  |
| Model 1 | 0.36 ± 0.28 | 0.35 ± 0.57 | 0.35 ± 0.40 | 0.787 | 0.963 | 1.03 ± 0.25 | 1.01 ± 0.24 | 1.01 ± 0.23 | 0.710 | 0.850 |
| Model 2 | 0.37 ± 0.29 | 0.35 ± 0.59 | 0.35 ± 0.30 | 0.258 | 0.992 | 1.03 ± 0.24 | 1.01 ± 0.23 | 1.02 ±0.22 | 0.821 | 0.317 |

*: Changes of fat mass percentage (Δ FM%) was annual change of FM% multiplied 6.2 years. Annual change of FM% represented as the coefficients of linear regression between FM% and follow-up duration multiplied by 6.2-y. Data analysis were conducted across tertiles of serum TMAO.

Data in model 1 were analyzed for the crude *P*-value across the tertiles of serum TMAO.

Data in model 2 were analyzed for the adjusted *P*-value across the tertiles of serum TMAO after adjusted covariates of baseline age, BMI, dietary intake (energy, protein and fat), daily activity and lifestyles (smoking, alcohol drinking), TC, TG, HDL-C, LDL-C, serum choline and betaine.

*P*_1_: Mean in each serum TMAO tertile was compared by ANOVA or ANCOVA for *P*_1_ to compare differences of total or regional Δ FM% across serum TMAO tertiles.

*P*_2_: Median of each serum TMAO tertile was applied in the linear regression model for *P*_trend_ to investigate the dose-response relationship of total and regional Δ FM% across the tertiles of serum TMAO.

**Table S3** **Linear regression analysis for the** **associations between total or regional** **Δ FD indices and per SD increase of serum TMAO ***

|  | **Women** | | **Men** | |
| --- | --- | --- | --- | --- |
|  | ***β* (SE)** | ***P*** | ***β* (SE)** | ***P*** |
| Δ Total FLR | 0.20 (0.14) | 0.147 | 0.32 (0.18) | 0.081 |
| Δ Trunk FLR | 0.26 (0.12) | **0.030** | -0.12 (0.19) | 0.522 |
| Δ Leg FLR | -0.21 (0.15) | 0.154 | -0.25 (0.21) | 0.229 |
| Δ Arm FLR | -0.42 (0.57) | 0.460 | 1.68 (1.44) | 0.244 |
| Δ Trunk-to-leg fat ratio | 0.07 (0.13) | **0.024** | -0.06(0.19) | 0.770 |

Changes of fat mass distribution indices (Δ FD indices) during the mean 6.2-y follow up, including total and regional Δ FLR as well as the Δ TLR, were annual Δ FD multiplied by 6.2-y. Annual Δ FD was represented as the coefficients from linear regressions of FD indices and follow-up duration. Data are shown as the coefficients (standard errors).

*: Multiple linear regression models were applied to investigate the association of Δ FD indices with per SD unit increase of serum TMAO. Data in models were adjusted for baseline age, BMI, dietary intake (energy, protein and fat), daily activity and lifestyles (smoking, alcohol drinking), diseases (CVD, type 2 diabetes), TC, TG, HDL-C, LDL-C, serum choline and betaine.

Abbreviations: TMAO, trimethylamine N-oxide; Δ FLR, changes of fat-mass to lean-mass ratio; Δ TLR: changes of trunk-to-leg fat ratio; SD, standard deviation

**Table S4 Sensitive analysis of total and regional Δ FD indices over 6.2-y among serum TMAO tertiles***

|  | **Women** | | | | | | | **Men** | | | | |
| --- | --- | --- | --- | --- | --- | --- | --- | --- | --- | --- | --- | --- |
|  | **Tertile 1**  **(N = 283)** | | **Tertile 2**  **(N = 282)** | | **Tertile 3**  **(N = 277)** | ***P*_1_** | ***P*_2_** | **Tertile 1**  **(N = 119)** | **Tertile 2**  **(N = 114)** | **Tertile 3**  **(N = 110)** | ***P*_1_** | ***P*_2_** |
| TMAO (μmol/L) | 0.80 ± 0.44 | | 1.65 ± 0.70 | | 4.61 ± 2.76 |  |  | 0.80 ± 0.42 | 1.71 ± 0.61 | 4.35 ± 2.32 |  |  |
| Δ Total FLR | |  | |  | |  |  |  |  |  |  |  |
| Model 1 | 1.13 ± 5.54 | | 1.28 ± 4.50 | | 1.91 ± 4.64 | 0.066 | 0.250 | 0.09 ± 4.04 | 1.13 ± 3.90 | 1.84 ± 4.24 | 0.147 | 0.369 |
| Model 2 | 1.12 ± 5.65 | | 1.30 ± 4.55 | | 1.88 ± 4.70 | 0.100 | 0.207 | -0.03 ± 4.02 | 1.09 ± 3.99 | 1.74 ± 4.22 | 0.150 | 0.555 |
| Δ Trunk FLR | |  | |  | |  |  |  |  |  |  |  |
| Model 1 | 0.62 ± 4.31 | | 0.90 ± 4.00 | | 1.66 ± 4.20 | **0.011** | 0.057 | 0.23 ± 4.11 | 0.93 ± 3.87 | 0.92 ± 4.18 | 0.770 | 0.745 |
| Model 2 | 0.67 ± 4.29 | | 0.89 ± 4.04 | | 1.68 ± 4.26 | **0.019** | 0.183 | 0.13 ± 4.12 | 0.77 ± 3.90 | 0.82 ± 4.24 | 0.330 | 0.662 |
| Δ Leg FLR |  | |  | |  |  |  |  |  |  |  |  |
| Model 1 | 0.73 ± 4.69 | | 1.48 ± 5.02 | | 0.91 ± 4.99 | 0.661 | 0.446 | 1.53 ± 4.45 | 2.20 ± 3.91 | 1.96 ± 4.84 | 0.506 | 0.139 |
| Model 2 | 0.76 ± 4.78 | | 1.51 ± 5.05 | | 0.48 ± 5.32 | 0.731 | 0.252 | 1.41 ± 4.43 | 2.26 ± 3.90 | 1.89 ± 4.97 | 0.715 | 0.960 |
| Δ Arm FLR |  | |  | |  |  |  |  |  |  |  |  |
| Model 1 | -0.39 ± 7.90 | | -2.57 ± 8.71 | | -1.46 ± 7.72 | 0.357 | 0.299 | -3.84 ± 4.61 | -2.75 ± 2.37 | 0.31 ± 8.75 | 0.300 | 0.703 |
| Model 2 | -0.27 ± 7.98 | | -2.68 ± 9.90 | | -1.48 ± 8.33 | 0.170 | 0.338 | -4.07 ± 4.11 | -2.92 ± 2.01 | 0.33 ± 9.18 | 0.160 | 0.776 |
| Δ Trunk-to-leg fat ratio | | | | | |  |  |  |  |  |  |  |
| Model 1 | 0.02 ± 0.23 | | 0.02 ± 0.24 | | 0.06 ± 0.23 | 0.210 | 0.477 | -0.24 ± 0.35 | -0.19 ± 0.29 | -0.21 ± 0.31 | 0.831 | 0.602 |
| Model 2 | 0.02 ± 0.23 | | 0.03 ± 0.23 | | 0.07 ± 0.24 | 0.087 | 0.080 | -0.24 ± 0.36 | -0.22 ± 0.29 | -0.22 ± 0.31 | 0.057 | 0.683 |

*: Data of participants younger than 65 years with no missing covariates were analyzed in sensitivity analysis. Δ FD indices, including total and regional Δ fat-mass to lean-mass ratio (Δ FLR) as well as the Δ trunk-to-leg fat ratio (Δ TLR), were annual changes of fat distribution multiplied by 6.2-y. Annual changes of fat distribution was represented as the coefficients from linear regressions of fat distribution indices and follow-up duration.

Data in model 1 were analyzed for the crude *P*-value across the tertiles of serum TMAO.

Data in model 2 were analyzed for the adjusted *P*-value across the tertiles of serum TMAO after adjusted covariates of baseline age, BMI, dietary intake (energy, protein and fat), daily activity and lifestyles (smoking, alcohol drinking), TC, TG, HDL-C, LDL-C, serum choline and betaine.

*P*_1_: Mean in each serum TMAO tertile was compared by ANOVA or ANCOVA for *P*_1_ to compare differences of total or regional ΔFD indices across serum TMAO tertiles.

*P*_2_: Median of each serum TMAO tertile was applied in the linear regression model for *P*_trend_ to investigate the dose-response relationship of total and regional ΔFD indices across the tertiles of serum TMAO.

**Table S5 Sensitive analysis of multivariable linear mixed-effects models of Δ FD indices over 6.2-y with serum TMAO**

|  | **Women** | ***P*** | **Men** | ***P*** |
| --- | --- | --- | --- | --- |
| Total FLR (ref. Tertile 1) |  |  |  |  |
| Tertile2 | -0.54 (-2.64, 1.56) | 0.615 | -0.74 (-4.57, 3.09) | 0.706 |
| Tertile3 | 2.50 (-0.41, 4.58) | 0.019 | 1.62 (-2.25, 5.49) | 0.413 |
| Time | -46.66 (-47.60, -45.13) | <0.001 | 1.36 (-0.59, 3.30) | <0.001 |
| Tertile2*Time | 0.80 (-1.33, 2.93) | 0.461 | 0.39 (-2.03, 2.81) | 0.753 |
| Tertile3*Time | -1.32 (-2.64, 1.01) | 0.052 | -0.56 (-3.00, 1.87) | 0.650 |
| Trunk FLR (ref. Tertile 1) |  |  |  |  |
| Tertile2 | 0.80 (-1.48, 3.08) | 0.490 | -2.62 (-2.85, 3.38) | 0.869 |
| Tertile3 | **2.91 (0.64, 5.19)** | **0.012** | 2.23 (-0.92, 5.37) | 0.166 |
| Time | 29.45 (28.40, 30.50) | <0.001 | -5.61 (-7.19, -4.03) | <0.001 |
| Tertile2*Time | -0.59 (-2.04, 0.85) | 0.420 | -1.29 (-2.09, 1.85) | 0.898 |
| Tertile3*Time | 1.30 (-0.17, 2.76) | 0.086 | -1.18 (-3.16, 0.80) | 0.242 |
| Leg FLR (ref. Tertile 1) |  |  |  |  |
| Tertile2 | -2.34 (-6.60, 1.92) | 0.282 | -1.12(-5.11, 2.87) | 0.418 |
| Tertile3 | 0.92 (-5.23, 3.39) | 0.676 | 1.96 (-2.07 5.99) | 0.552 |
| Time | 0.17 (-2.03, 2.36) | <0.001 | 2.02 (0.01, 4.05) | <0.001 |
| Tertile2*Time | 1.22 (-1.49, 3.92) | 0.378 | 0.59 (-1.93, 3.11) | 0.657 |
| Tertile3*Time | 0.97 (-1.77, 3.71) | 0.489 | -0.79 (-3.32, 1.75) | 0.517 |
| Arm FLR (ref. Tertile 1) |  |  |  |  |
| Tertile2 | -1.30 (-7.20, 4.59) | 0.665 | -1.85 (-6.25, 2.55) | 0.410 |
| Tertile3 | 0.32 (-5.64, 6.28) | 0.916 | 1.60 (-2.84, 6.04) | 0.481 |
| Time | 6.30 (3.26, 9.35) | <0.001 | 3.71 (1.48, 5.95) | <0.001 |
| Tertile2*Time | 1.07 (-2.67, 4.82) | 0.574 | 0.74 (-2.04, 3.52) | 0.601 |
| Tertile3*Time | 1.07 (-2.73, 4.86) | 0.581 | -0.78 (-3.57, 2.02) | 0.586 |
| Trunk-to-leg fat ratio (ref. Tertile 1) |  |  |  |  |
| Tertile2 | -0.002 (-0.05, 0.05) | 0.925 | 0.83 (-0.08,0.25) | 0.321 |
| Tertile3 | -0.02 (-0.07, 0.03) | 0.346 | 0.14 (-0.15, 0.18) | 0.871 |
| Time | 0.58 (0.56, 0.60) | <0.001 | -0.14 (-0.22, -0.06) | <0.001 |
| Tertile2*Time | 0.01 (-0.02, 0.04) | 0.373 | -0.02 (-0.13, 0.08) | 0.619 |
| Tertile3*Time | **0.02 (1e-03, 0.06)** | **0.041** | -0.01 (-0.10,0.11) | 0.884 |

Data of participants who younger than 65 years with no missing covariates were assessed in the linear mixed-effects model, with serum TMAO, time, and the serum TMAO × time interaction term as fixed effects and the subject identifier as a random intercept.

All models were adjusted for baseline age, BMI, dietary intake (energy, protein and fat), daily activity and lifestyles (smoking, alcohol drinking), diseases (CVD, type 2 diabetes), TC, TG, HDL-C, LDL-C, serum choline and betaine.

Abbreviations: LMEMs: Linear mixed-effects model; FLR, fat-mass to lean-mass ratio; TLR: trunk-to-leg fat ratio; TMAO, trimethylamine N-oxide

**Table S6 Comparison of baseline characteristics between the included and excluded population**

|  | **Included** | **Excluded** | ***P*** |
| --- | --- | --- | --- |
| N | 1964 | 1198 |  |
| Age (years)^1^ | 57.4 ± 4.9 | 57.7 ± 5.6 | 0.101 |
| Sex (n, %)^3^ | 1421 (72.4) | 842 (70.3) | 0.211 |
| Marriage (n, %)^3^ | 1756 (90.6) | 1067 (89.4） | 0.306 |
| Monthly income (Yuan/person)^3^ |  |  | **0.040** |
| <3000 | 1536 (80.0) | 975 (83.0) |  |
| >=3000 | 384 (20.0) | 199 (17.0) |  |
| Menopausal state^3^ (n, %)^3^ | 1151 (62.9) | 813 (61.1) | 0.308 |
| Dietary intakes |  |  |  |
| Energy (kcal/d)^2^ | 1759.6 (1490.6, 2129.4) | 1737.6 (1439.7, 2134.6) | 0.266 |
| Protein (g/d)^2^ | 73.6 (59.9, 90.7) | 74.2 (57.6, 91.7) | 0.640 |
| Fat (g/d)^2^ | 57.5 (44.2, 75.4) | 56.7 (42.2, 74.4) | 0.624 |
| No Smoking (n, %)^3^ | 1702 (86.6) | 984 (82.4) | **0.001** |
| No Alcohol drinking (n, %)^3^ | 1863 (94.8) | 1105 (92.6) | **0.004** |
| Tea drinking (n, %)^3^ | 972 (50.1) | 582 (48.8) | 0.465 |
| Daily activity (MET)^2^ | 36.5 (30.5,58.2) | 36.4 (30.1, 57.9) | 0.624 |
| CVDs (n, %)^3^ | 194 (11.5) | 136 (12.9) | 0.349 |
| No Type 2 diabetes (n, %)^3^ | 417 (90.5) | 281 (88.9) | 0.516 |
| No Hypertension (n, %)^3^ | 347 (75.3) | 232 (73.4) | 0.559 |
| Biochemical indicators |  |  |  |
| TC, mmol/L^2^ | 5.4 (4.8,6.1) | 5.3 (4.6,6.1) | 0.174 |
| HDL_C, mmol/L^2^ | 1.4 (1.2, 1.6) | 1.4 (1.3, 1.6) | 0.964 |
| LDL_C, mmol/L^2^ | 3.6 (3.0, 4.2) | 3.6 (3.0, 4.2) | 0.519 |
| TG, mmol/L^2^ | 1.3 (0.9,1.8) | 1.3 (0.9,1.8) | 0.110 |
| Fasting Glu, mmol/L^2^ | 4.6 (4.2,5.0) | 4.6 (4.2,5.1) | 0.095 |

^1^: Continue data which was normally distributed was displayed as mean with SD. T-test was conducted for the *P*-value.

^2^: Continue data which was not normally distributed was displayed as median with IQR. Kruskal-Wallis was conducted for the *P*-value.

^3^: Categorical data was displayed as frequency and percentage. 𝜒2 tests was conducted for the *P*-value.

TMAO, trimethylamine N-oxide; BMI, body mass index; WHR, waist-to-hip ratio; CVDs, cardiovascular diseases; TC, total cholesterol; HDL-C, high density leptin cholesterol; LDL-C, low density leptin cholesterol; TG, Triglycericies; MET, metabolic equivalent-h/d; IQR, interquartile range.
